# Depth and the Uncertainty of Statistical Knowledge on Musical Creativity Fluctuate Over a Composer's Lifetime

**DOI:** 10.3389/fncom.2019.00027

**Published:** 2019-04-30

**Authors:** Tatsuya Daikoku

**Affiliations:** Department of Neuropsychology, Max Planck Institute for Human Cognitive and Brain Sciences, Leipzig, Germany

**Keywords:** statistical learning, entropy, mutual information, information theory, Markovian, Bayesian, order, n-gram

## Abstract

Brain models music as a hierarchy of dynamical systems that encode probability distributions and complexity (i.e., entropy and uncertainty). Through musical experience over lifetime, a human is intrinsically motivated in optimizing the internalized probabilistic model for efficient information processing and the uncertainty resolution, which has been regarded as rewords. Human's behavior, however, appears to be not necessarily directing to efficiency but sometimes act inefficiently in order to explore a maximum rewards of uncertainty resolution. Previous studies suggest that the drive for novelty seeking behavior (high uncertain phenomenon) reflects human's curiosity, and that the curiosity rewards encourage humans to create and learn new regularities. That is to say, although brain generally minimizes uncertainty of music structure, we sometimes derive pleasure from music with uncertain structure due to curiosity for novelty seeking behavior by which we anticipate the resolution of uncertainty. Few studies, however, investigated how curiosity for uncertain and novelty seeking behavior modulates musical creativity. The present study investigated how the probabilistic model and the uncertainty in music fluctuate over a composer's lifetime (all of the 32 piano sonatas by Ludwig van Beethoven). In the late periods of the composer's lifetime, the transitional probabilities (TPs) of sequential patterns that ubiquitously appear in all of his music (familiar phrase) were decreased, whereas the uncertainties of the whole structure were increased. Furthermore, these findings were prominent in higher-, rather than lower-, order models of TP distribution. This may suggest that the higher-order probabilistic model is susceptible to experience and psychological phenomena over the composer's lifetime. The present study first suggested the fluctuation of uncertainty of musical structure over a composer's lifetime. It is suggested that human's curiosity for uncertain and novelty seeking behavior may modulate optimization and creativity in human's brain.

## Introduction

### Statistical Learning and Uncertainty in the Brain

The brain models external phenomena as a hierarchy of dynamical systems that encode probability distributions and complexity (i.e., entropy and uncertainty) over states in the world. Based on the internalized hidden model, it can predict a future state and optimize behavior and action to resolve the uncertainty (Friston, 2010). Within predictive-coding framework, this behavior mandates the suppression of prediction errors (prediction of content) and uncertainty (prediction of the context or precision of predictability and uncertainty) through updating internal model that generates predictions and the belief (Kanai et al., 2015). It has been considered that aesthetic appreciation of music can be modulated by these brain function: Through musical experience over lifetime, a human is intrinsically motivated in optimizing the internalized probabilistic model for efficient information processing and the uncertainty resolution, which has been regarded as rewords. For example, previous studies demonstrated that the precise prediction (Przysinda et al., 2017) and uncertainty perception (Hansen and Pearce, 2014) in music is stronger in proficient musicians than non-musicians.

This generative model could cover statistical learning (SL) theory of brain (Saffran et al., 1996; Cleeremans et al., 1998; Perruchet and Pacton, 2006). The SL is an implicit process by which the brain automatically calculates the statistical distribution of sequential phenomena based on Bayesian inference (Daikoku et al., 2012, 2014, 2016, 2017a, 2018; Yumoto and Daikoku, 2016; Daikoku and Yumoto, 2017), grasps the uncertainty (Hasson, 2017), predicts a future state based on the internal statistical model, and optimize action for achieving a given goal (Monroy et al., 2017a,b). By SL, generation of culture (Feher et al., 2016), individuality of creativity (Daikoku, 2018b) can be originated. Although brain tries to realize valuable behaviors at the lowest possible informational cost and uncertainty, it also seeks slightly suboptimal solution if the solution can be afforded at a significantly low uncertainty (Tishby and Polani, 2011). In other words, human's behavior appears to be not necessarily directing to efficiency but sometimes act unefficiently to explore a maximum rewards of uncertainty resolution. Previous studies suggest that the drive for novelty seeking behavior (high uncertain phenomenon) reflects human's curiosity and that the curiosity rewards encourage humans to create and learn new regularities (Kagan, 1972; Wittmann et al., 2008; Krebs et al., 2009; Schwartenbeck et al., 2013). Furthermore, a certain degree of uncertainty generates excitement and pleasure (Shen et al., 2015) because we explore a maximum curiosity rewards. Although brain generally minimizes prediction errors and uncertainty (Friston, 2010), we sometimes derive pleasure from prediction errors under conditions such as enjoying music listening due to curiosity and motivation for novelty seeking behavior by which we anticipate the resolution of uncertainty. Some literatures propose the hypothesis that the recurrent resolution of uncertainty activates reward networks that underwrite pleasure induced by listening to music (Koelsch, 2014; Salimpoor et al., 2015). It has been suggested that creativity can be explained as by-products of such intrinsic curiosity rewards (Schmidhuber, 2006). That is, human seems to look for some forms of optimality between uncertain and certain situations through action by which we are expected a maximum curiosity rewards, and hence our action gives rise to increasing as well as decreasing uncertainty. Recent studies imply that the curiosity rewards encourage humans to create and learn new regularities (Schmidhuber, 2006), and the fluctuations in uncertainty of predictions could contribute to aesthetic appreciation of art and music (Koelsch, 2014). Thus, it is hypothesized that human's intrinsic curiosity and motivation may modulate optimization and efficiency of prediction and action involved in SL. Recent computational studies on music suggest that, from early to late periods in the composer's lifetime, the transitional probabilities (TPs) of familiar phrase that ubiquitously appears in all of his music were gradually decreased (Daikoku, 2018d). This suggests that the statistical knowledge (Daikoku, 2018a) may be susceptible to long-term experience that modulates brain's probabilistic model (Hansen and Pearce, 2014). A neurophysiological study also suggested that sequences with higher entropy were learned based on higher-order TP, whereas those with lower entropy were learned based on lower-order TP (Daikoku et al., 2017b; Daikoku and Yumoto, 2019). Another study suggested that certain regions or networks perform specific computations of entropy (i.e., uncertainty), which are different from TP (i.e., prediction) of each content (Hasson, 2017). Thus, interaction between prediction and uncertainty in perceptive systems is an important topic to understand whole process of brain SL in both computational and neurophysiological areas (Daikoku, 2018c; Yumoto and Daikoku, 2018). Nevertheless, to our knowledge, few study examined relationships between SL, uncertainty and musical creativity and how curiosity for uncertain and novelty seeking behavior modulates musical creativity. The present study investigated how the probabilistic model and the uncertainty in music fluctuate over a composer's lifetime (all of the 32 piano sonatas by Ludwig van Beethoven).

### Computational Model

The computational model and simulation have been used to understand SL systems (e.g., Pearce and Wiggins, 2012; Rohrmeier and Rebuschat, 2012; Daikoku, 2018a; Wiggins, 2018; Daikoku and Yumoto, 2019). Although experimental approaches are necessary for understanding the real-world brain's function, the modeling approaches partially outperform experimental results under conditions that are impossible to replicate in an experimental approach (e.g., long-term statistical variation over the decades within a person and across cultures) and serves an important dual role in providing a quantitative account of observed empirical effects and in generating novel predictions to guide empirical research (e.g., Elman, 1990; Thiessen et al., 2013; Carreiras et al., 2014). Computational modeling can also express the relevant neural networks and neural hardware of sensory cortices (Turk-browne et al., 2009; Roux and Uhlhaas, 2014). For example, simple recurrent network (SRN), which is classified as a neural network and was firstly devised by Elmer Elman (1990), learns sequential co-occurrence statistics by error-driven learning in which the gap between the prediction of a next input and the actual input drives changes to the weights on its internal connections. The SRN (Rogers and McClelland, 2004) and a modified SRN (Altmann, 1999; Dienes et al., 1999) implement a similarity space in which words referring to similar objects or actions were located more closely to one another than to words referring to dissimilar objects or actions. The neural network and deep learning such as Long-Short Term Memory (LSTM) (Hochreiter and Urgen Schmidhuber, 1997), on the other hand, is not intended to be a model of the relationship between human episodic and semantic memory although they proceed in this direction. Corpus-based approaches such as hyperspace analog to language (HAL) (Lund and Burgess, 1996), bound encoding of the aggregate language environment (BEAGLE) (Jones and Mewhort, 2007), Latent Semantic Analysis (LSA) (Landauer and Dumais, 1997) are based on abstraction of episodic memory of input information and encoding in a multidimensional semantic space as semantic memory. Their models could also generate semantic similarity spaces in the similar way. For instance, when a verb of “drink” occurs, the models predict subsequent words that can be drunken. PARSER (Perruchet and Vinter, 1998), Competitive Chunker (Servan-Schreiber and Anderson, 1990), Information Dynamics of Music (IDyOM) (Pearce and Wiggins, 2012), Information Dynamics of Thinking (IDyOT) (Wiggins, 2018), and other *Markovian* models including the n-gram and nth-order Markov models (Daikoku, 2018b), can implement chunking hypotheses that learning is based on extracting, storing, and combining small chunks. Particularly, information-theoretical models including *Markovian* processes have been applied to neurophysiological studies of SL in human brain as well as computational simulation (Pearce et al., 2010a; Pearce and Wiggins, 2012; Daikoku et al., 2014, 2016, 2017a, 2018; Yumoto and Daikoku, 2016, 2018; Daikoku and Yumoto, 2017; Daikoku, 2018c). These neurophysiological experiments showed consistent evidence: neural activities for stimuli with high information content (i.e., low probability) are larger than those with low information content (i.e., high probability). Furthermore, these SL effects were larger when humans are exposed stimulus sequence with less information entropy (uncertainty), compared with when they are exposed stimulus sequence with high information entropy (Daikoku et al., 2017c). The mutual information of information theory, which has been assumed as the reduction of uncertainty afforded by observations (see section Mutual Information of nth-order SL model), is also correlated with neuronal activity in limbic cortex (Harrison et al., 2006). This neural phenomenon is in agreement with a *Bayesian* hypothesis in theoretical neurobiology that the brain encodes probabilities (beliefs) about the causes of sensory data, and that these beliefs are updated in response to new sensory evidence based on Bayesian inference (Kersten et al., 2004; Knill and Pouget, 2004; Doya et al., 2007; Friston, 2010; O'Reilly et al., 2012; Parr and Friston, 2018; Parr et al., 2018). Formulations of self-organization (Karl Friston, 2013; Kirchhoff et al., 2018) and brain connectivity (Parr and Friston, 2018) are also expressed using an information-theoretical concept called Markov blankets (Pearl, 1988). The blanket of a state is the only knowledge necessary to predict the behavior of that state and the adjacent state. If we know everything within a blanket, knowledge about things outside the blanket becomes uninformative about things inside the blanket. For example, Parr and Friston (2018) hypothesized that a neuronal population reflecting a given variable only need receive connections from those populations representing its blanket and explained this notion from perception, planning, attention, and movement. The Markov blanket may also represent in part chunk formation although it's not sufficient. Markov decision process (MDP) (Schwartenbeck et al., 2013; Karl Friston et al., 2014, 2015; Pezzulo et al., 2015), which has often been used for reinforcement learning in AI and robotics, extends the simple perceptive process by adding active process (controlling predictability by choice, called “*policy*”) and “*rewards*” (giving motivation). The IDyOM is also an extension of Markov model to precisely modeling SL of musical sequence combining several concomitant information such as pitch, duration, onset, scale degree, and so on. The SL based on IDyOM could also be reflected in neurophysiological responses within the predictive-coding framework (Pearce et al., 2010b). The IDyOT also takes advantage of information theory to represent domain-general SL mechanisms that cover both language and music (Wiggins, 2018). Particularly, this model implements semantic and episodic memory systems, and captures hierarchical SL process from lower- to higher-level using boundary entropy: spectrum of auditory sequence is chunked into phonemes, then morphemes, then words (Wiggins, in press). In summary, information-theoretical models including Markovian processes can capture a variety of neurophysiological phenomena on SL such as prediction, uncertainty, a part of chunk formation, and policy of action, across domains, and modality.

A previous study reported that SL effects based on TPs occurs action as well as perception (Monroy et al., 2017c). This suggests that SL also contributes production of sequences. In other words, from psychological perspective, TP distribution sampled from music based on Markov models may also refer to the characteristics of a composer's statistical knowledge: a high-probability transition in music may be one that a composer is more likely to predict and choose based on the latest n states, compared to a low-probability transition. Thus, the Markov model is used in the interdisciplinary realms of neuroscience, behavioral science, engineering, and informatics. A computational study using nth-order Markov or n-gram models suggested that time-course variations of statistics in music reflect time-course variations of a composer's statistical knowledge (Daikoku, 2018d). Neurophysiological studies also suggested that time-course variations of statistics of auditory sequence modulate SL effects (Daikoku et al., 2017c) and that the SL effects of sequences with higher entropy were lower than those with lower entropy, even when TP itself is same between these two sequences (Daikoku et al., 2017c). These studies suggest that time-course variations of TPs and entropy may partially be able to predict the SL model in human's brain.

### Mathematical Interpretation of Brain's Statistical Learning Based on Information Theory

#### Nth-Order Transitional Probability

According to SL theory, the brain automatically computes both lower- and higher-order TPs in sequences (Furl et al., 2011; Yumoto and Daikoku, 2016, 2018, grasps uncertainty/entropy in the whole sequences Hasson, 2017, and predicts a future state based on the internalized statistical model Friston, 2010. The TP is a conditional probability of an event B given that the latest event A has occurred, written as P(B|A). The nth-order TP distributions sampled from sequential information such as music and language can be expressed by nth-order Markov models. The nth-order Markov model is based on the conditional probability of an event e_n+1_, given the preceding *n* events based on Bayes' theorem (*P(e*_*n*+1_*|e*_*n*_*)*). From psychological perspective, the conditional probability (*P(e*_*n*+1_*|e*_*n*_*)*) can be interpreted as positing that the brain predicts a subsequent event e_n+1_ based on the preceding events e_n_ in a sequence. In other words, learners expect the event with the highest TP based on the latest n states, whereas they are likely to be surprised by an event with lower TP. Furthermore, TPs are often translated as information contents (IC) (–*log*_2_*1/P(e*_*n*+1_*|e*_*n*_*)*) of information theory (Pearce and Wiggins, 2006). The lower IC (i.e., higher TPs) means higher predictabilities and smaller surprising, whereas the higher IC (i.e., lower TPs) means lower predictabilities and larger surprising. In the end, a tone with lower IC may be one that a composer is more likely to predict and choose as a next tone, compared to tones with higher IC. IC can be used in computational studies of music to discuss psychological phenomena involved in prediction and SL.

#### Entropy and Uncertainty

**Figure 1:**
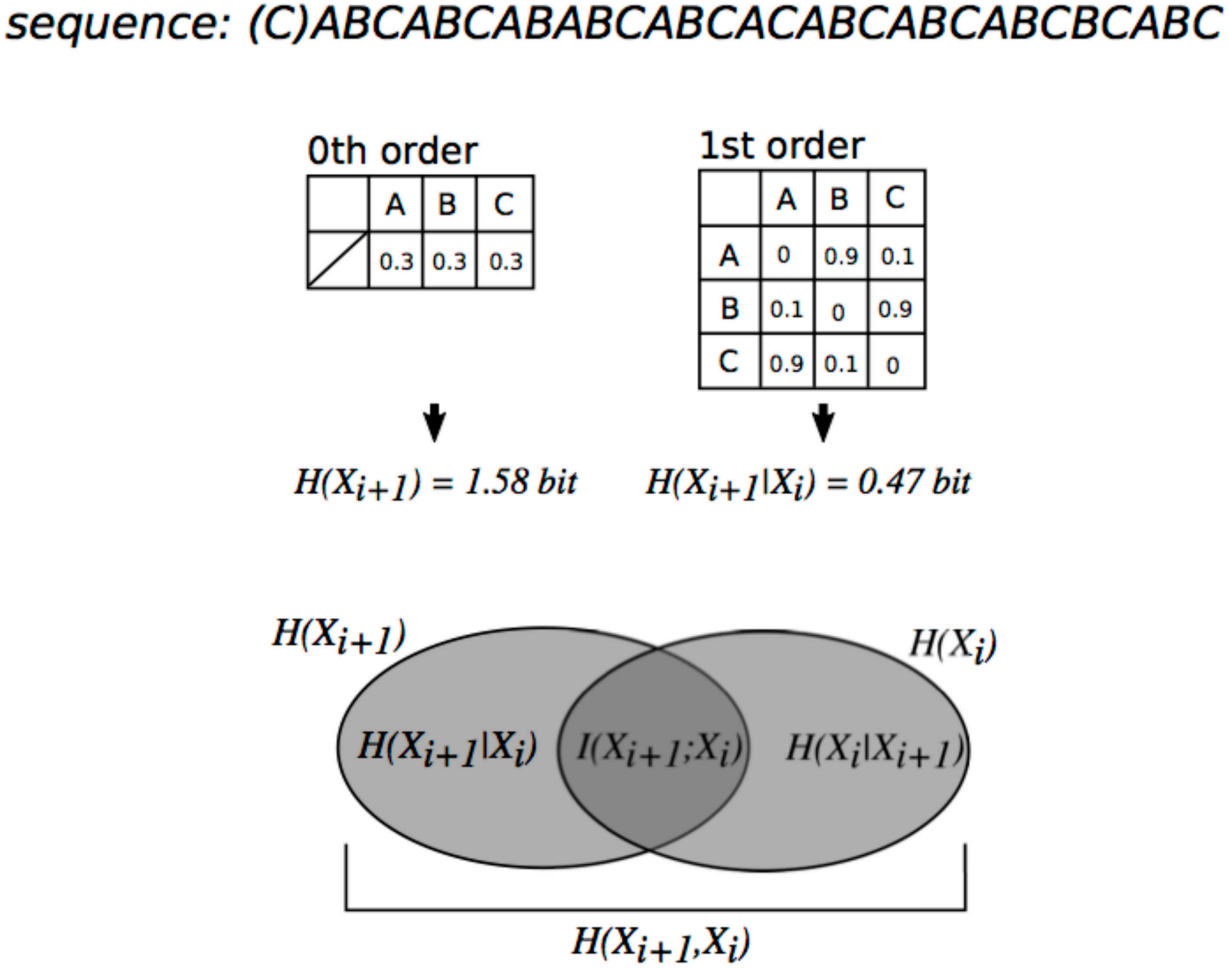
Relationship between order of transitional probabilities, conditional entropy, and mutual information illustrated using a Venn diagram. The degree of dependence on X_i_ for X_i+1_ is measured by mutual information [mutual information (*I(X;Y)*] = entropy (*H(X*_*i*+1_*)*)–conditional entropy (*H(X*_*i*+1_*|X*_*i*_*)*)]. The mutual information of sequences in this figure is more than 0. Thus, each event X_i+1_ in the sequence is dependent on a preceding event *X*_*i*_.

Entropy as well as TP of each event is used to understand predictability of a sequence (Pearce, 2005). Entropy (e.g., see Figure 1) is calculated from probability distribution, interpreted as uncertainty (Friston, 2010), and used to evaluate neurophysiological effects of uncertainty in SL (Harrison et al., 2006) and curiosity (Loewenstein, 1994). A previous study reported that neural systems of uncertainty perception were partially independent of those of prediction of each content (Hasson, 2017). Some articles, however, suggest that uncertainty modulates predictability of each content in SL (Daikoku et al., 2017c). Furthermore, uncertainty of auditory and visual statistics is coded by modality-general, as well as modality-specific, neural systems (Strange et al., 2005; Nastase et al., 2014). This suggests that the neural basis that codes uncertainty as well as prediction, is a domain-general system. Thus, there seems to be neural and psychological interactions of perceptions between prediction and uncertainty.

#### Mutual Information of nth-order SL Model

Mutual information (MI) and pointwise Mutual information (PMI) are a measure of the mutual dependence between the two variables. The PMI refers to each event in sequence (local dependence), whereas MI refers to the average of all events in the sequence (global dependence). In the framework of SL based on TPs (*P(e*_*n*+1_*|e*_*n*_*)*), MI explains how an event *e*_*n*+1_ is dependent on the preceding event *e*_*n*_. Thus, MI is a key to understanding order of SL. For instance, conventional oddball sequence, which consists of a frequent stimulus with high probability of appearance and a deviant stimulus with low probability of appearance, has weak dependence between two adjacent events (*e*_*n*_*, e*_*n*+1_) and shows low MI, because event *e*_*n*+1_ appears independently of preceding events *e*_*n*_. In contrast, SL sequence based on TPs, but not probabilities of appearance, has strong dependence between two adjacent events and shows larger MI. For example, typical SL paradigm that consists of concatenation of pseudo-words with three stimuli has large MI until 2nd-order Markov or tri-gram models [i.e., *P(C|AB)*)], whereas it has low MI from 3rd-oder Markov or four-gram models [i.e., *P(D|ABC)*)]. Thus, MI is sometimes used to evaluate hierarchical SL in both neurophysiological and computational studies (Harrison et al., 2006; Pearce et al., 2010b).

In this section, the three types of information-theoretical evaluation of SL models (i.e., IC, entropy, and MI) were explained from psychological aspects. In sum, (1) IC reflects surprising/predictability. A tone with lower IC (i.e., higher TPs) may be one that a composer is more likely to predict and choose as a next tone, compared to tones with higher IC. (2) Entropy reflects uncertainty of whole sequences. (3) MI reflects hierarchy of statistics and is interpreted as dependence of preceding sequential events in SL. Using them, the present study investigated how prediction, uncertainty, and the depth of implicit knowledge in music vary over a composer's lifetime (all of the 32 piano sonatas by Ludwig van Beethoven).

### Ludwig Van Beethoven's Piano Sonata

The German composer and pianist Ludwig van Beethoven (1770–1827) remains one of the most famous and influential of all composers. It is believed that his music strongly expresses the psychological variations and visions of his life (Sullivan, 1927; Boucourechliev, 1963). Beethoven's compositional career is often divided into the early (around 1802), middle (around 1802–1814), and late periods (from about 1814) (Dahlhaus, 1991; Adorno-Wiesengrund, 1993). It is generally thought that his works in the early period were strongly influenced by his predecessors in classicism, such as Wolfgang Amadeus Mozart (1756–1792) and Franz Joseph Haydn (1732–1809), whereas his works in the late period show his personal character and experience (Sullivan, 1927) and accompanying intellectual depth and personal expression (Dahlhaus, 1991; Adorno-Wiesengrund, 1993). Thus, his psychological variations on thinking and experience may form the statistical characteristics of his music that may reflect a composer's statistical knowledge (Johnson et al., 1985). It is believed that he always explored new directions of musical composition and gradually expanded his scope of music over his lifetime (Dahlhaus, 1991; Adorno-Wiesengrund, 1993). Using Beethoven's piano sonatas over his lifetime, the present study examined time-course variations of three types of statistics in music: TPs (ICs) of sequential patterns that appear in all 32 sonatas, entropy of whole TP distribution, and the MI. It was hypothesized that, because of his exploration of new directions in musical composition over his lifetime, TP of phrases that frequently appear in the early period (i.e., sequences with high TP) might decrease in the late period (i.e., decreasing TP), whereas entropy might increase in the late period. It would be very interesting if the psychological variations in which Beethoven explored new directions and gradually expanded his scope of music over his lifetime were reflected in the SL models of his music.

## Methods

**Figure 2:**
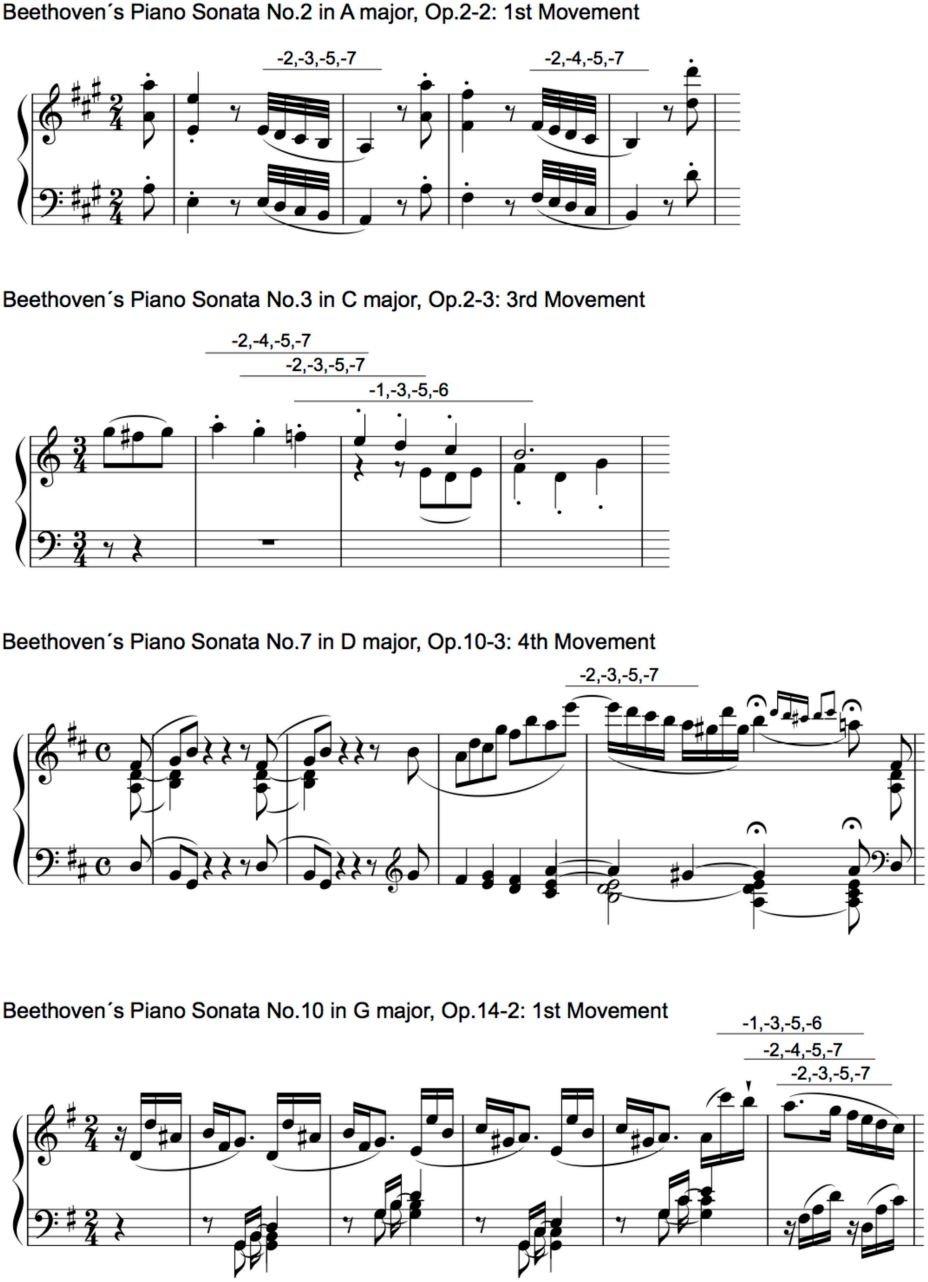
Representative sequences of [0,−2,−3,−5,−7], [0,−2,−4,−5,−7], and [0,−1,−3,−5,−6] in Beethoven's piano sonatas in the early period.

The Piano Sonata with all of its movements by Ludwig van Beethoven (No.1 in F minor, Op.2-1 to No.32 in C minor, Op.111, composed 1795–1822) was used in the present study. Using a scorewriter (Finale version 25, MI Seven Japan, Inc.), electronic scoring data of the sequences of highest pitch were extracted from the XML files. The highest pitches were chosen based on the following definitions: the highest pitches that can be played at a given point in time, the pitches with slurs can be counted as one, and the grace notes were excluded. Although melody is sometimes not highest pitches e.g., bass melodies), the present study only analyzed the highest pitch because different melodies could concurrently appear in some titles of music, and melody is often played in highest pitches. Using all the sequences of highest pitches in a movement of a Sonata, sequential patterns based on uni- to four-grams were extracted. For each type of the sequential patterns, all pitches were numbered so that the first pitch was 0 in each transition, and an increase or decrease in a semitone was 1 and −1 based on the first pitch, respectively. The representative examples were shown in Figure 2. This revealed interval patterns but not pitch pattern. This procedure was employed to eliminate the effects of the change of key on transitional patterns. The interpretation of the change of key depends on musicians, and it is difficult to define in an objective manner. Thus, the results in the present study may represent a variation of statistics associated with relative pitch rather than absolute pitch. Then, the TPs of the sequential patterns were calculated based on 0th- to 3rd-order Markov chains. Furthermore, TPs of all the movements in each piece of sonata (No.1 to No.32) were weighted averaged: an average in which probability of each phrase is multiplied by a weight before summing to a single average value. That is weightings are the equivalent of having that many like items with the same value involved in the average. The *n*th-order Markov chain is the conditional probability of an event *e*_*n*+1_, given the preceding event *e*_*n*_:

(1) $$\begin{array}{l} P\left(e_{n+1}|e_{n}\right)\;=\;\frac{P\left(e_{n+1}\;\cap \;e_{n}\right)}{P\left(e_{n}\right)} \end{array}$$

The ICs (*I*) and conditional entropy (*H*) in the nth-order TP distribution (hereafter, Markov entropy) were calculated using TPs in the framework of information theory:

(2) $$\begin{array}{lll} I\left(e_{n+1}|e_{n}\right)\; & = & \;-\log_{2}P\left(e_{n+1}|e_{n}\right)\;\left(bit\right) \end{array}$$

(3) $$\begin{array}{lll} H\left(B|A\right)\; & = & \;{-\sum }_{i}\;\underset{j}{\sum }P\left(ai\right)P\left(bj|ai\right)\;\log_{2}\;P\left(bj|ai\right)\;\left(bit\right) \end{array}$$

where *P(*bj|ai*)* is a conditional probability of sequence “*ai bj*.” Then, MI (*I(X;Y)*) were calculated in 1st-, 2nd-, and 3rd-order Markov models. MI is an information theoretic measure of dependency between two variables. From entropy values, the MI can also be expressed as

(4) $$\begin{array}{l} I\left(X;Y\right)=\;\underset{x,y}{\sum }p\left(x,y\right)\log\left(\frac{p\left(x,y\right)}{p\left(x\right)p\left(y\right)}\right)\\ \;=\;\underset{x,y}{\sum }p\left(x,y\right)\log\left(\frac{p\left(x,y\right)}{p\left(x\right)}\right)-\underset{x,y}{\sum }p\left(x,y\right)\log p\left(y\right)\\ \;=\;\underset{x,y}{\sum }p\left(x\right)p\left(y|x\right)\log p\left(y|x\right)-\underset{x,y}{\sum }\log p\left(y\right)p\left(x,y\right)\\ \;=\;\underset{x}{\sum }p\left(x\right)\left(\underset{y}{\sum }p\left(y|x\right)\log p\left(y|x\right)\right)\\ \;-\;\underset{y}{\sum }\log p\left(y\right)\left(\underset{x}{\sum }p\left(x,y\right)\right)\\ \;=\;-\underset{x}{\sum }p\left(x\right)H\left(Y|X=x\right)-\underset{y}{\sum }p\left(y\right)\log p\left(y\right)\\ \;=\;-H\left(Y|X\right)+\;H\left(Y\right)\\ \;=\;H\left(Y\right)-H\left(Y|X\right)\;\left(bit\right) \end{array}$$

where p(x,y) is the joint probability function of X and Y, p(x), and p(y) are the marginal probability distribution functions of X and Y respectively, H(X) and H(Y) are the marginal entropies, H(X|Y) and H(Y|X) are the conditional entropies, and H(X,Y) is the joint entropy of X and Y (Figure 1) (Daikoku, 2018a). Based on psychological and information-theoretical concepts, the Equation (4) can be regarded that the amount of entropy (uncertainty) remaining about Y after X is known. That is, the MI is corresponding to reduction in entropy (uncertainty). In each order of Markov models, the sequential patterns that ubiquitously appear in all 32 sonatas (hereafter, familiar phrase) were extracted. Then, TPs of the familiar phrases were averaged (0th: 20 phrases, 1st: 37 phrases, 2nd: 12 phrases, and 3rd: 3 phrases) (Table 1). The 32 sonatas were divided based on the well-known 3 periods: early (No.1 to 12, No19, and No20), middle (No.13 to 18 and No. 21 to 27), and late (No. 28 to 32). Then, I conducted analysis of variances (ANOVAs) with a within-subject factor order (0th vs. 1st vs. 2nd vs. 3rd) and a between-subjects factor composition period of the sonatas (early vs. middle vs. late) for the TPs of familiar phrases and entropy of whole music, and an ANOVA with a within-subject factor order (1st vs. 2nd vs. 3rd) and a between-subjects factor composition period (early vs. middle vs. late) for the mutual information. When we detected significant effects, Bonferroni-corrected *post-hoc* tests were conducted for further analysis. Then, in each order of Markov models, the TPs of familiar phrase and the uncertainty of whole music were compared by Pearson's correlation analysis. Statistical significance levels were set at *p* = 0.05 for all analyses.

**Table 1:** Sequential patterns that appear in all 32 sonatas (i.e., phrases) in each order of Markov models.

**Order**	**Sequential patterns**
0th	[−2], [−1], [1], [0], [2], [−3], [3], [5],[−4],[4],[−5], [12], [−7], [7], [9], [−12], [8], [−6], [6], [−9]
1st	[1,1], [−1,2], [−1,−1], [1,−2], [0,1], [3,3], [0,2], [1,−4], [0,3], [0,5], [1,−1], [−2,2], [−2,5]
2nd	[−2,−4,−5], [0,0,0], [−1,−3,−5], [2,4,5], [−2,−3,−5], [1,3,5], [2,3,5], [2,0,−1], [−2,−3,−2], [−1,0,2], [1,3,1], [−1,0,−1]
3rd	[−2,−3,−5,−7], [−2,−4,−5,−7], [−1,−3,−5,−6]

## Results

### TPs of Familiar Phrases

**Figure 3:**
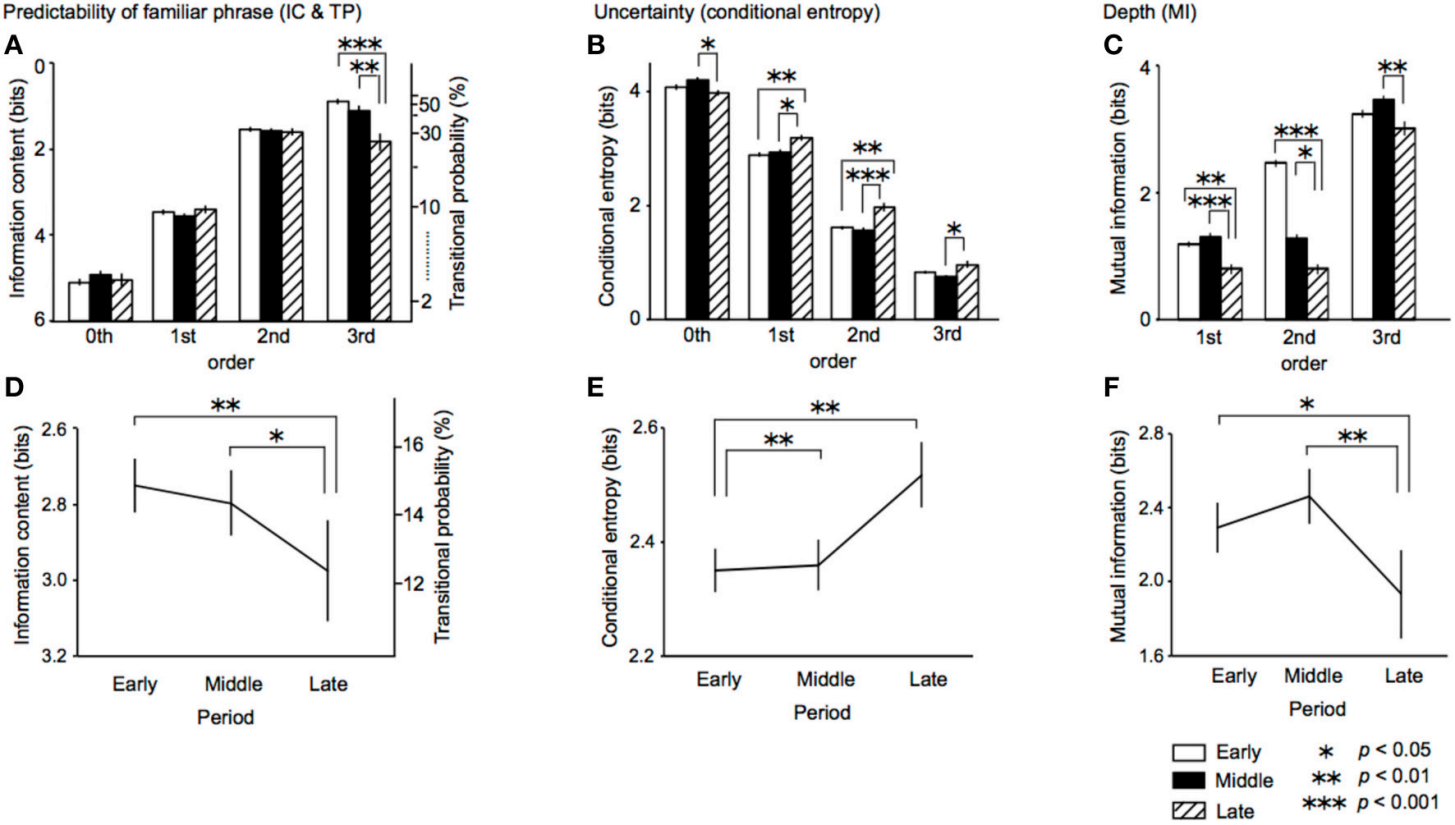
The period-order interactions **(A–C)** and main effects of period **(D–F)** in the ANOVA of ICs and TPs of familiar phrases, conditional entropy of TP distribution, and the depth of implicit knowledge (MI) in the early (opened bars), middle (filled bars), and late (dashed bars) periods. IC, information content; TP, transitional probability; MI, mutual information.

In the TPs of familiar phrases, An ANOVA with a within-subject factor order (0th vs. 1st vs. 2nd vs. 3rd) and a between-subjects factor composition period (early vs. middle vs. late) was conducted. As a result, the main effect of period was significant [*F*(2, 29) = 6.02, *p* = 0.007, partialη^2^ = 0.29, early > late, *p* = 0.005; middle > late, *p* = 0.032] (Figure 3D). The period-order interaction was also significant [*F*(6) = 6.82, *p* < 0.001, partialη^2^ = 0.32] (Figure 3A). The 3rd-order TPs in late period were significantly lower than those in early (*p* < 0.001) and middle periods (*p* = 0.003). That is to say, the 3rd-order TPs of familiar phrases in the late period only decrease during lifetime. The main effect of order was significant [*F*(3, 87) = 1108.35, *p* < 0.001, partialη^2^ = 0.98]. The 0th-order TPs were significantly lower than the 1st-, 2nd-, and 3rd-order TPs (all: *p* < 0.001). The 1st-order TPs were significantly lower than the 2nd-, 3rd-order TPs (all: *p* < 0.001). The 2nd-order TPs were significantly lower than the 3rd-order TPs (*p* = 0.007).

### Entropy and Uncertainty

In entropy of whole TP distribution, ANOVA with a within-subject factor order and a between-subjects factor composition period of sonatas was performed. The main effect of period was significant [*F*_(2, 29)_ = 7.58, *p* = 0.002, partialη^2^ = 0.34, early < middle, *p* = 0.005; early < late, *p* = 0.002] (Figure 3E). The period-order interaction was also significant [*F*(6) = 6.68, *p* < 0.001, partialη^2^ = 0.32] (Figure 3B). The entropies of 0th-order TPs in late period were significantly lower than those in the middle periods (*p* = 0.034). The entropies of 1st-order TPs in late period were significantly higher than those in the early (*p* = 0.004) and middle periods (*p* = 0.014). The entropies of 2nd-order TPs in late period were significantly higher than those in the early (*p* = 0.001) and middle periods (*p* < 0.001). The entropies of 3rd-order TPs in late period were significantly higher than those in the middle periods (*p* = 0.017). The main effect of order was significant [*F*(1.73, 50.30) = 2329.84, *p* < 0.001, partialη^2^ = 0.99]. The entropies of 0th-order TPs were significantly lower than the 1st-, 2nd-, and 3rd-order TPs (all: *p* < 0.001). The entropies of 1st-order TPs were significantly lower than the 2nd-, 3rd-order TPs (all: *p* < 0.001). The entropies of 2nd-order TPs were significantly lower than the 3rd-order TPs (*p* = 0.007).

### Hierarchy of Statistics: Mutual Information

In the mutual information, an ANOVA with a within-subject factor order and a between-subjects factor composition period was conducted. The main effect of period was significant [*F*(2, 29) = 9.08, *p* = 0.001, partialη^2^ = 0.39, early > late, *p* = 0.020; middle > late, *p* = 0.001] (Figure 3F). The period-order interaction was also significant [*F*_(4)_ = 2.80, *p* = 0.034, partialη^2^ = 0.16] (Figure 3C). The mutual information of 1st- and 2nd-order TPs in late period were significantly lower than those in the early (1st: *p* = 0.004; 2nd: *p* = 0.012) and middle periods (1st: *p* < 0.001; 2nd: *p* < 0.001). The mutual information of 3rd-order TPs in late period was significantly higher than those in the middle periods (*p* = 0.008). The main effect of order was significant [*F*_(1.32, 38.16)_ = 2350.56, *p* < 0.001, partialη ^2^ = 0.99]. The mutual information of 1st-order TPs were significantly lower than the 2nd-, 3rd-order TPs (all: *p* < 0.001). The 2nd-order TPs were significantly lower than the 3rd-order TPs (*p* < 0.001).

## Discussion

Brain encodes probability distributions and the entropy/uncertainty of musical information (Koelsch et al., 2018) and mandates the suppression of prediction errors and uncertainty by updating the internal probabilistic model of music that generates predictions and the belief (Kanai et al., 2015). In other words, through musical experience over lifetime, a human generally tries to optimize the internalized probabilistic model for efficient information processing and the uncertainty resolution, which has been regarded as rewords. On the other hand, to explore the maximum rewards of uncertainty resolution, human's behavior appears to be not necessarily directing to efficiency, but sometimes be drove by unefficient, uncertain, and novelty information, which is thought as curiosity (Kagan, 1972; Wittmann et al., 2008; Krebs et al., 2009; Schwartenbeck et al., 2013). Thus, although brain typically minimizes uncertainty of music structure, we sometimes derive pleasure from music with uncertain structure due to curiosity for novelty-seeking behavior by which we anticipate further rewords by uncertainty resolution. The present study, using all the Beethoven's piano sonatas over his lifetime, examined how the probabilistic model and the uncertainty in music fluctuate over a composer's lifetime. The transitional probability and information content (TP), information content (IC), conditional entropy, and mutual information (MI) can be calculated based on *n*th-order Markov models. Based on psychological and neurophysiological studies on SL (Harrison et al., 2006; Pearce et al., 2010b; de Zubicaray et al., 2013; Daikoku et al., 2015; Monroy et al., 2017c), these three information can be translated to psychological indices: a tone with lower IC (i.e., higher TPs) may be one that a composer is more likely to choose as a next tone, compared to tones with higher IC, whereas the entropy and MI are interpreted as uncertainty and the order of the SL, respectively. It was hypothesized that probability, uncertainty, and the order of SL models is fluctuated over Beethoven's lifetime. If so, it may suggest that his curiosity for uncertain and novelty seeking behavior modulate optimization and creativity in human's brain.

The TPs of familiar phrase (i.e., sequences that appear in all 32 sonatas) were decreased in the late periods (Figure 3D), whereas the entropies in music were increased in the late periods (Figure 3E). In other words, there was no significant difference between early and middle periods, while there was significant difference between middle and late periods. Particularly, the 3rd-order TPs in the late period decrease during lifetime (Figure 3A). According to musicological studies, his works in the early period were strongly influenced by his predecessors in classicism whereas his works in the late period show his personal character and experience (Sullivan, 1927). It is believed that he always explored new directions of musical composition and gradually expanded his scope of music over his lifetime (Dahlhaus, 1991; Adorno-Wiesengrund, 1993). The findings of the present study may suggest the hypothesis that the psychological variations over lifetime are reflected in the statistical structure in music. The decreasing of the TPs of familiar phrase and increasing of the entropies may imply that, in the late period, he tried novel composition strategies in which he avoided familiar sequences in the early period, and tried various transitional patterns by which nth-order TPs are broadly distributed. The previous study detected time-course variation of predictability of familiar phrases over his lifetime (Daikoku, 2018d). The present study, furthermore, suggested that there seems to be interactions between prediction and uncertainty.

The decreasing of TPs of familiar phrase over Beethoven's lifetime was obvious in the higher-, but not lower-, order models (Figure 3A). This may suggest that a higher-, rather than lower-, order statistical structure reflects specific statistical knowledge that is susceptible to experience and novelty seeking behavior. The entropy (i.e., uncertainty) of TP distribution may also support the hypothesis (Figure 3B). The entropies of higher-order (1st to 3rd), but not lower-order (0th) models in late period increased compared with those in early period. Furthermore, MI in late periods was lower compared with those early and middle periods (Figure 3F). This suggests that, in the late period, each event of tone hardly depends on preceding successive events of tones. Typical Western-classical music has strict syntactic rules based on music theory. Therefore, a forthcoming tone can partially be predicted from preceding successive tones based on the rules. According to previous studies, syntax of musical sequences is partially expressed by conditional probabilities (Rohrmeier and Cross, 2008), although it is not sufficient to account for all of the music syntax. The findings on MI in this study may suggest that, in the late period, the composer avoided a tone that can easily be predicted based on typical transition rules involved in music syntax.

In sum, the present study detected time-course variation of predictability of familiar phrases, uncertainty of whole music, and the depth of SL in music that were composed over Beethoven's lifetime. According to corpus studies, the historical characteristics of music can be extracted based on the era (e.g., Albrecht and Huron, 2014; Gjerdingen, 2014; White, 2014). This indicates that strategies of composition and musical knowledge depend on the era. The present study also suggests that the characteristics of music can be extracted based on the periods within a composer's lifetime. In addition, the higher-order hierarchical structure showed larger time-course variations of both predictability of familiar phrases and uncertainty of whole music. From the psychological perspective, it would be interesting if the higher- (i.e., deep), rather than lower-order statistical knowledge was susceptible to experience and novelty seeking behavior. The present study also suggested that there are interactions between prediction and uncertainty. It is of note, however, that the present study did not directly investigate the composer's statistical knowledge of music, as only the statistics of musical scores were analyzed. Furthermore, the present study only analyzed one composer, therefore could not discuss universal phenomenon on SL. This suggests that there may be other possible explanations for the findings of the present study. For instance, it might have been Beethoven's plan to compose the sonatas from familiar and lower entropy to unfamiliar and larger entropy based on the statistical structure of music. Future study should investigate SL of music from many composers using interdisciplinary approaches in parallel.

## Conclusion

The present study investigated how predictability of familiar phrases that was used in all of music, uncertainty of whole structure, and the order of the probabilistic models in music fluctuates over a composer's lifetime, and discussed the results from psychological perspective within SL framework. The results suggest that the higher-, rather than lower-order statistical knowledge may be susceptible to experience and novelty seeking behavior. The present study also suggested that there might be interactions between prediction and uncertainty. The present study first suggested that uncertainty may be increased in a composer's lifetime, and that the higher-order probabilistic model may be susceptible to experience and novelty seeking behavior over the composer's lifetime. It is suggested that human's curiosity for uncertain and novelty seeking behavior may modulate creativity in human's brain, and that the fluctuations of uncertainty could reflect aesthetic appreciation of music. To more understand brain's predictive function, future study is needed to examine relationships between prediction of familiar phrases and uncertainty perception, using both modeling and experimental approaches in parallel.

## Author Contributions

The methodology of the present study and were considered by the author. The author analyzed all of the data and prepared the figures, and wrote the manuscript text.

### Conflict of Interest Statement

The author declares that the research was conducted in the absence of any commercial or financial relationships that could be construed as a potential conflict of interest.
